# Multi-omics analysis of immune-related microbiome and prognostic model in head and neck squamous cell carcinoma

**DOI:** 10.1007/s00784-024-05645-y

**Published:** 2024-04-20

**Authors:** Yingqiao Liu, Haitao Lin, Weijun Zhong, Yudi Zeng, Guihai Zhou, Zhifeng Chen, Shi Huang, Leitao Zhang, Xiqiang Liu

**Affiliations:** 1 Department of Oral and Maxillofacial Surgery, Nanfang Hospital, Southern Medical University, Guangzhou, 510515 China; 2 Faculty of Dentistry, The University of Hong Kong, Hong Kong, Hong Kong SAR China

**Keywords:** Head and neck squamous cell carcinoma, Immune subtype, Microbiome, Multi-omics, Prognosis

## Abstract

**Objectives:**

The aim of our study is to explore the transcriptional and microbial characteristics of head and neck cancer’s immune phenotypes using a multi-omics approach.

**Materials and methods:**

Employing TCGA data, we analyzed head and neck squamous cell carcinoma (HNSCC) immune cells with CIBERSORT and identified differentially expressed genes using DESeq2. Microbial profiles, obtained from the TCMA database, were analyzed using LEfSe algorithm to identify differential microbes in immune cell infiltration (ICI) subgroups. Random Forest algorithm and deep neural network (DNN) were employed to select microbial features and developed a prognosis model.

**Results:**

We categorized HNSCC into three immune subtypes, finding ICI-2 with the worst prognosis and distinct microbial diversity. Our immune-related microbiome (IRM) model outperformed the TNM staging model in predicting survival, linking higher IRM model scores with poorer prognosis, and demonstrating clinical utility over TNM staging. Patients categorized as low-risk by the IRM model showed higher sensitivity to cisplatin and sorafenib treatments.

**Conclusions:**

This study offers a comprehensive exploration of the ICI landscape in HNSCC. We provide a detailed scenario of immune regulation in HNSCC and report a correlation between differing ICI patterns, intratumor microbiome, and prognosis. This research aids in identifying prime candidates for optimizing treatment strategies in HNSCC.

**Clinical relevance:**

This study revealed the microbial signatures associated with immunophenotyping of HNSCC and further found the microbial signatures associated with prognosis. The prognostic model based on IRM microbes is helpful for early prediction of patient prognosis and assisting clinical decision-making.

**Supplementary Information:**

The online version contains supplementary material available at 10.1007/s00784-024-05645-y.

## Introduction

Head and neck cancer (HNC), ranking as the sixth most common cancer globally, accounts for approximately 30,000 fatalities each year [1]. The predominant pathological subtype of HNC is squamous cell carcinoma [2]. In advanced cases of head and neck squamous cell carcinoma (HNSCC), the primary causes of mortality are local recurrence, cervical lymph node metastases, and resistance to conventional chemotherapy, which often leads to treatment failure. In HNSCC, the tumor microenvironment (TME) is composed of altered tumor cells, immune cells, and stromal cellular components [3]. Extensive research on TME has highlighted the pivotal role of tumor-infiltrating immune cells in tumor progression, recurrence, metastasis, and response to immunotherapy treatments [4].

The role of microbiota as a key regulator in immune cell activation, inflammation, and cancer progression has been increasingly recognized. Specifically, microbiota influences these biological processes through mechanisms involving nuclear factor kappa B (NF-κB), type I interferon, and inflammasome activation [5]. Moreover, the local immune system’s interaction with gut microbiota plays a crucial role in modulating immune responses, tissue damage, and the development of cancer, as evidenced by several studies [6]. The emerging role of microbes in cancer research is increasingly becoming a focal point, offering profound insights into cancer development [7, 8]. In addition to their intrinsic role, microbes are also being explored as a potential tool for adjunct diagnosis in cancer research [9, 10]. Studies have shown that patients with higher loads of *Fusobacterium nucleatum* (*F. nucleatum*) DNA in cancerous tissues tend to have shorter survival durations, highlighting its potential as a biomarker for prognosis [11]. The newly released Cancer Microbiome Atlas (TCMA) encompasses curated microbial profiles from a comprehensive collection of 3,689 samples across 1,772 patients, spanning five The Cancer Genome Atlas (TCGA) programs and 21 anatomical locations [12]. This atlas has been actively utilized in research on various cancers, including gastric, colon, and HNSCC. Its application facilitates multi-omics studies, enabling systematic analyses of microbe-host interactions [13].

To elucidate the impact of various factors on the formation and maintenance of the TME, as well as on clinical prognosis in HNSCC, we analyzed TME infiltration patterns using multi-omics data from the TCGA HNSCC cohort. This included correlating immune status with genetic and intratumor immune-related microbiome characteristics. Our findings provide valuable insights into the immunological and microbial landscape of HNSCC, and their impacts on patients’ prognosis. These findings have important implications for enhancing treatment strategies and improving patient outcomes.

## Materials and methods

### Data acquisition and immune cell profiling in HNSCC

We acquired RNA sequencing data and corresponding clinical profiles for HNSCC from the TCGA database. Specifically, gene expression profiles of TCGA-HNSC in FPKM format were retrieved from the TCGA portal. To analyze these profiles, we employed CIBERSORT, which generated a fractional matrix estimating the abundances of 22 distinct immune cell types, providing insights into the immune cell infiltration landscape within the TME.

### Identifying gene features linked to immune cell infiltration subgroups

To identify Differentially Expressed Genes (DEGs) related to immune cell infiltration (ICI) subgroups, we employed the DESeq2 package in R (version 4.3.1) [14]. We considered genes with false discovery rates (FDR) less than 0.05 and absolute fold changes greater than 2 as significant, selecting them for subsequent analyses. For gene set enrichment analysis, we utilized the KEGG REST API (https://www.kegg.jp/kegg/rest/keggapi.html) to obtain the latest gene annotations of KEGG Pathways. These annotations served as the background dataset against which genes were mapped. The enrichment analysis was then conducted using the R package ‘clusterProfiler’ (version 3.14.3), allowing us to determine the enriched gene sets [15].

### Microbial abundance profiling in TCGA samples

The normalized microbial abundance profiles were obtained from the TCMA database, which reanalyzed whole-genome and transcriptome sequencing data from treatment-naive TCGA samples to quantify microbial reads. A total of 153 samples, accompanied by RNA sequencing and clinical profiles, were selected for further analysis. We employed the Lefse algorithm to identify differential microbes among the three ICI subgroups, considering microbes with adjusted P values less than 0.05 as significantly different in abundance. Alpha diversity was assessed using the R package ‘vegan’, while beta diversity was measured through Bray-Curtis distances. Principal-coordinate analysis (PCoA) plots, created with the R packages ‘GUniFrac’ and ‘ggplot2’, visualized these diversity measures.

### Microbial feature selection and model evaluation in survival analysis

To distinguish between long and short survival patients, we utilized the Random Forest (RF) algorithm, implemented via the ‘caret’ package in R, for selecting microbial features [16]. Optimal feature selection was achieved using the ‘randomForest’ R package, resulting in the identification of six discriminatory microbes from the top-performing model. We trained this model using 70% of the sample set, and 30% of the sample set were used as testing set. A deep neural network (DNN) was employed to establish the model utilizing the training dataset. During the training phase, each neuron modifies the sum of its weighted inputs through the application of a sigmoid function. The input layer is comprised of chosen variables. The hidden layer processes and weights this input data, while the output neuron delivers a prediction regarding the prognostic risk for a HNSCC patient. The model’s performance was evaluated using the Receiver Operating Characteristic (ROC) curve, generated by the ‘pROC’ R package, with the Area Under the Curve (AUC) metric assessing the model’s discriminative capability [17]. We employed the ‘pRRophetic’ package in R to predict drug sensitivity across our sample set. ‘pRRophetic’ estimates the half-maximal inhibitory concentration (IC50) of drugs, leveraging gene expression data to predict the response to various chemotherapeutic agents [18].

## Results

### HNSCC patients could be classified as three immune subtypes

**Fig. 1:**
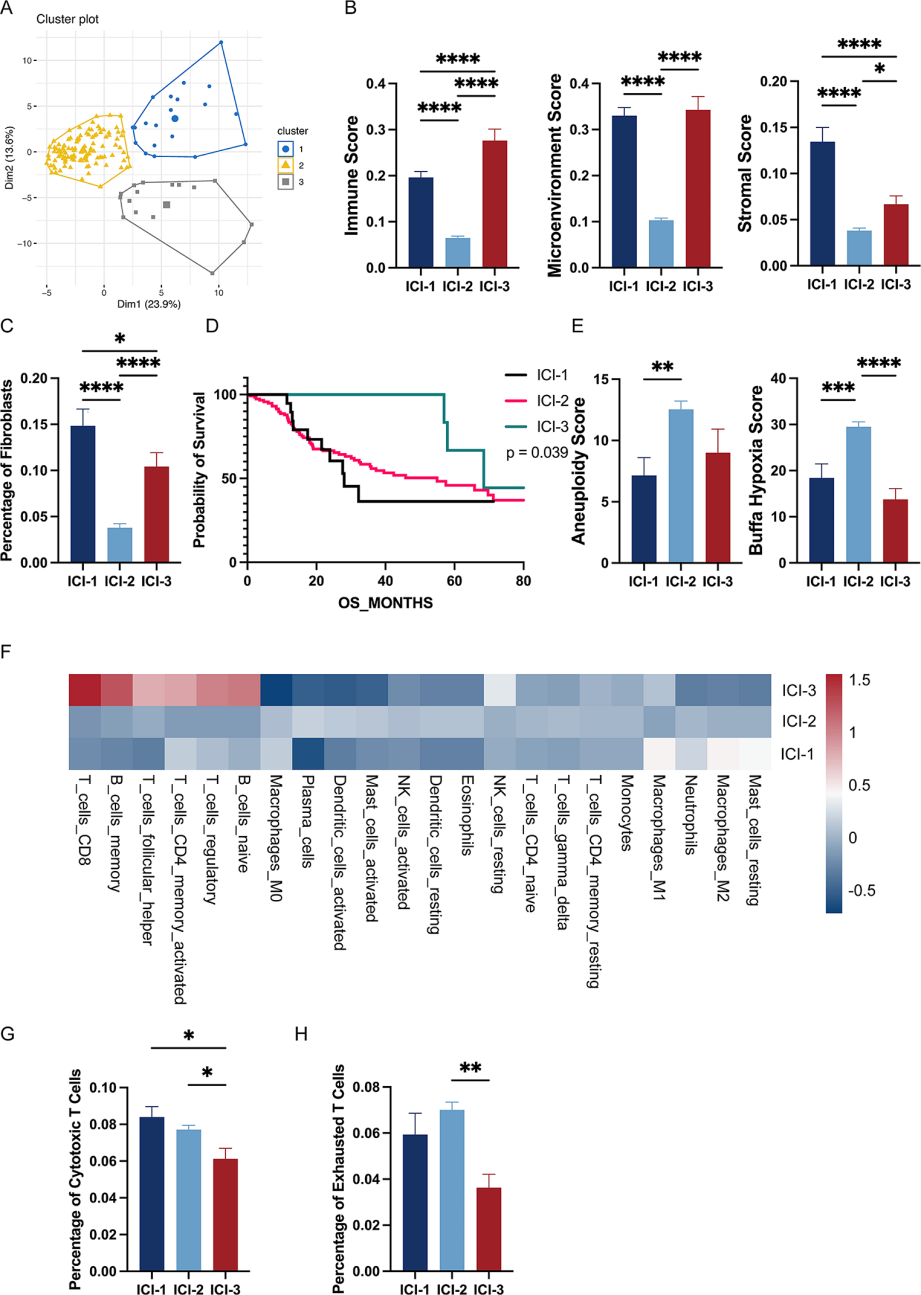
HNSCC Patients Could Be Classified As Three Immune Subtypes.(**A**) Principal Component Analysis (PCA) of Immune Cell Infiltration Subtypes in HNSCC. Each point represents an individual HNSCC sample, color-coded according to the identified immune subtype: blue for ICI-1, yellow for ICI-2, and grey for ICI-3. (**B**) Comparative Analysis of Immune, Microenvironment, and Stromal Scores Across ICI Subtypes. (**C**) Fibroblast Proportion in Immune Cell Infiltration Subtypes of HNSCC. (**D**) Kaplan-Meier Survival Curves for ICI Subtypes in HNSCC. Each line represents one of the subtypes: ICI-1 (black line), ICI-2 (red line), and ICI-3 (green line) with p-value of 0.039. (**E**) Aneuploidy Scores and Buffa Hypoxia Scores Across Immune Cell Infiltration Subtypes in HNSCC. (**F**) Heatmap of Immune Cell Proportions in ICI Subtypes of HNSCC. Each column in the heatmap represents a different type of immune cell, while each row corresponds to one of the ICI subtypes. The color intensity within the heatmap indicates the relative proportion of each immune cell type within a particular ICI subtype, ranging from low (blue) to high (red) proportions. (**G**) Cytotoxic T Cells Proportion in Immune Cell Infiltration Subtypes of HNSCC. (**H**) Exhausted T Cells Proportion in Immune Cell Infiltration Subtypes of HNSCC. Each bar represents the average value of a specific group, with error bars indicating the standard error of the mean (SEM). Statistical differences between groups are denoted by asterisks above the corresponding bars, based on One-Way ANOVA (*p < 0.05, **p < 0.01, ***p < 0.001, **** *p* < 0.0001)

In the TCMA dataset, we analyzed 152 HNSCC patients with whole-genome sequencing (WGS) data. Utilizing CIBERSORT and xCell algorithms on their transcriptome data, we quantified immune cell proportions, immune scores, microenvironment scores, and stroma scores for each patient [19, 20]. Subsequent cluster analysis of immune cell infiltration identified three distinct immune subtypes: ICI-1, ICI-2, and ICI-3 (Fig. 1A). In the analysis of the three immune subtypes, it was observed that ICI-2 exhibits the lowest immune score, in contrast to ICI-3, which demonstrates the highest. Notably, while the immune score of ICI-1 is intermediate, positioned between ICI-2 and ICI-3, this subtype is distinguished by having the highest stroma score (Fig. 1B). This is particularly marked by a pronounced abundance of fibroblasts (Fig. 1C). In this analysis, the ICI-3 category is associated with the longest Overall Survival (OS) and the best prognosis among the three classifications studied (Fig. 1D). A higher proportion of patients in the local advanced stage are observed to fall within the ICI-2 category. Notably, in our dataset, all patients classified under ICI-3, which is characterized by the best prognosis, are HPV positive (Table 1). Furthermore, a comparative analysis reveals that ICI-2 is distinguished by elevated Aneuploidy Scores and Buffa Hypoxia Scores relative to the other two categories (Fig. 1E). In immune cell infiltration, ICI-3 distinctively exhibits a higher proportion of several cell types: CD8 T cells, memory B cells, T follicular helper (Tfh) cells, activated CD4 memory T cells, regulatory T (Treg) cells, and naive B cells (Fig. 1F). This unique cellular composition highlight ICI-3 has positive immune response regulation contributing better prognosis. Although ICI-2 was characterized by a substantial presence of cytotoxic T cells (Fig. 1G), there was a notably higher proportion of exhausted T cells in this subgroup (Fig. 1H). This finding further substantiated that ICI-2 represents an immunosuppressive type.

**Table 1:** Clinical Characteristics of ICI Subtypes

Characteristics	ICI-1(*N* = 20)	ICI-2(*N* = 116)	ICI-3(*N* = 16)	Total(*N* = 152)	*p*-value
**Clinical Stage**					0.045
STAGE IV	12(9.38%)	58(45.31%)	2(1.56%)	72(56.25%)	
STAGE III	2(1.56%)	18(14.06%)	2(1.56%)	22(17.19%)	
STAGE II	1(0.78%)	20(15.63%)	3(2.34%)	24(18.75%)	
STAGE I	4(3.13%)	5(3.91%)	1(0.78%)	10(7.81%)	
**Histologic Grade**					< 0.001
G3-4	5(3.38%)	27(18.24%)	11(7.43%)	43(29.05%)	
G1-2	15(10.14%)	86(58.11%)	4(2.70%)	105(70.95%)	
**HPV Status**					< 0.001
HPV-	19(12.84%)	91(61.49%)	0(0.00%)	110(74.32%)	
HPV+	1(0.68%)	21(14.19%)	16(10.81%)	38(25.68%)	
**N Stage**					0.920
N3	0(0.00%)	3(2.52%)	0(0.00%)	3(2.52%)	
N2	8(6.72%)	35(29.41%)	2(1.68%)	45(37.82%)	
N1	2(1.68%)	13(10.92%)	1(0.84%)	16(13.45%)	
N0	9(7.56%)	41(34.45%)	5(4.20%)	55(46.22%)	
**T Stage**					< 0.001
T4	7(5.34%)	41(31.30%)	0(0.00%)	48(36.64%)	
T3	4(3.05%)	24(18.32%)	1(0.76%)	29(22.14%)	
T2	2(1.53%)	30(22.90%)	7(5.34%)	39(29.77%)	
T1	7(5.34%)	6(4.58%)	2(1.53%)	15(11.45%)	

Clinical stage, N stage and T stage were according to the TNM classification

### Comprehensive transcriptomic profiling of ICI subtypes

**Fig. 2:**
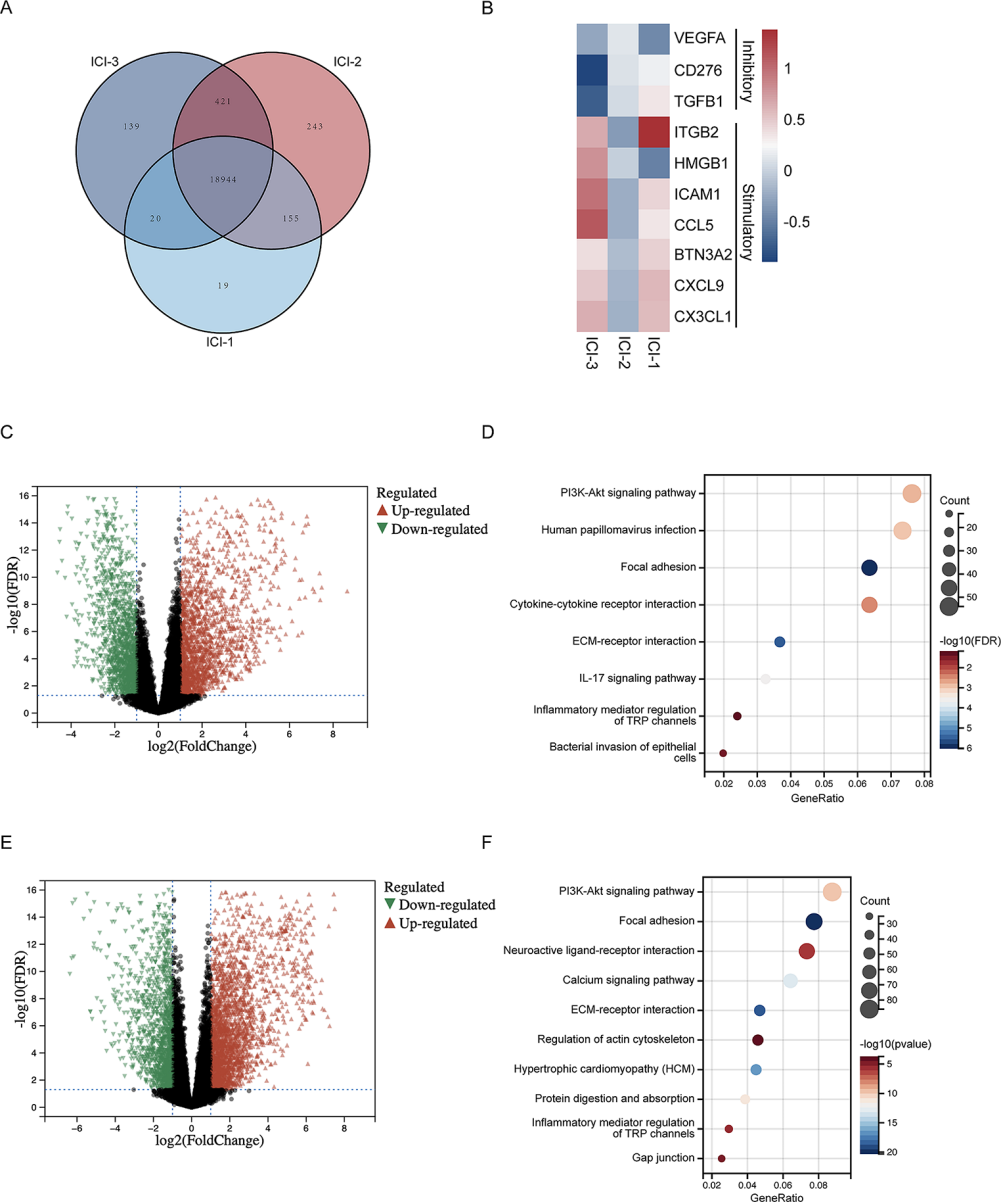
Comprehensive Transcriptomic Profiling of ICI Subtypes. (**A**) Venn Diagram of Unique Gene Expressions in ICI Subtypes. The diagram highlights the number of uniquely expressed genes in each subtype: 19 in ICI-1, 243 in ICI-2, and 139 in ICI-3. The overlapping and non-overlapping regions of the circles represent shared and unique genes, respectively. (**B**) Heatmap of Top 10 Differentially Expressed Immune-Regulatory Genes in ICI Subtypes. Each row in the heatmap represents one of the immune-regulatory genes, while the columns correspond to the ICI subtypes. The color intensity in the heatmap indicates the expression level of each gene in each subtype, ranging from low (blue) to high (red) expression. (**C**) Volcano Plot of Differentially Expressed Genes between ICI-2 and ICI-3. The plot identifies genes with a fold change of 2 and a false discovery rate (FDR) of 0.05. Genes that are upregulated in ICI-2 compared to ICI-3 are represented in red, while those downregulated are shown in green. (**D**) Bubble Chart of KEGG Pathway Enrichment for Upregulated Genes in ICI-2 Compared to ICI-3. Each bubble represents a KEGG pathway, with the size indicating the level of enrichment and the color representing the significance of the enrichment (*p* < 0.05). (**E**) Volcano Plot of Differentially Expressed Genes between ICI-1 and ICI-3. The plot identifies genes with a fold change of 2 and a false discovery rate (FDR) of 0.05. Genes that are upregulated in ICI-1 compared to ICI-3 are represented in red, while those downregulated are shown in green. (**F**) Bubble Chart of KEGG Pathway Enrichment for Upregulated Genes in ICI-1 Compared to ICI-3. Each bubble represents a KEGG pathway, with the size indicating the level of enrichment and the color representing the significance of the enrichment (*p* < 0.05)

To investigate the differences among these three immune phenotypes, we conducted an in-depth analysis of their transcriptomes. Utilizing a Venn diagram, we identified unique and shared genes across the three ICI subtypes. Our findings revealed 19, 243, and 139 unique genes in ICI-1, ICI-2, and ICI-3, respectively (Fig. 2A). Among the top 10 significantly enriched immune-regulatory genes, those categorized as immune-stimulatory were predominantly upregulated in ICI-3, while genes identified as immune-inhibitory were downregulated in this subtype (Fig. 2B). A comparative analysis between ICI-2 and ICI-3 demonstrated a significant disparity in gene expression, with 1,941 genes upregulated and 1,647 downregulated in ICI-2 (Fig. 2C). Further, KEGG pathway analysis of the upregulated genes in ICI-2 indicated a predominant enrichment in pathways associated with infection, including ‘Human papillomavirus infection’, ‘Cytokine-cytokine receptor interaction’, ‘IL-17 signaling pathway’, ‘Inflammatory mediator regulation of TRP channels’, and ‘Bacterial invasion of epithelial cells’ (Fig. 2D). This enrichment suggests a potential link between microbial presence and altered immune microenvironment, possibly contributing to the observed poorer prognosis in ICI-2. In the differential gene analysis between ICI-1 and ICI-3, we identified 2,534 genes upregulated in ICI-1 (Fig. 2E). These genes predominantly enrich pathways related to cell-matrix and cell-cell interactions, notably in ‘Focal adhesion’ and ‘ECM-receptor interaction’ (Fig. 2F). This suggests a significant role of these pathways in the distinct immune phenotype of ICI-1. In the TME, in addition to the immune responses induced by the tumor cells themselves, the presence of microbes cannot be ignored. It is precisely these findings that have inspired us to explore the composition and abundance differences of these microbes.

### Immune-related microbial features of ICI subtypes

**Fig. 3:**
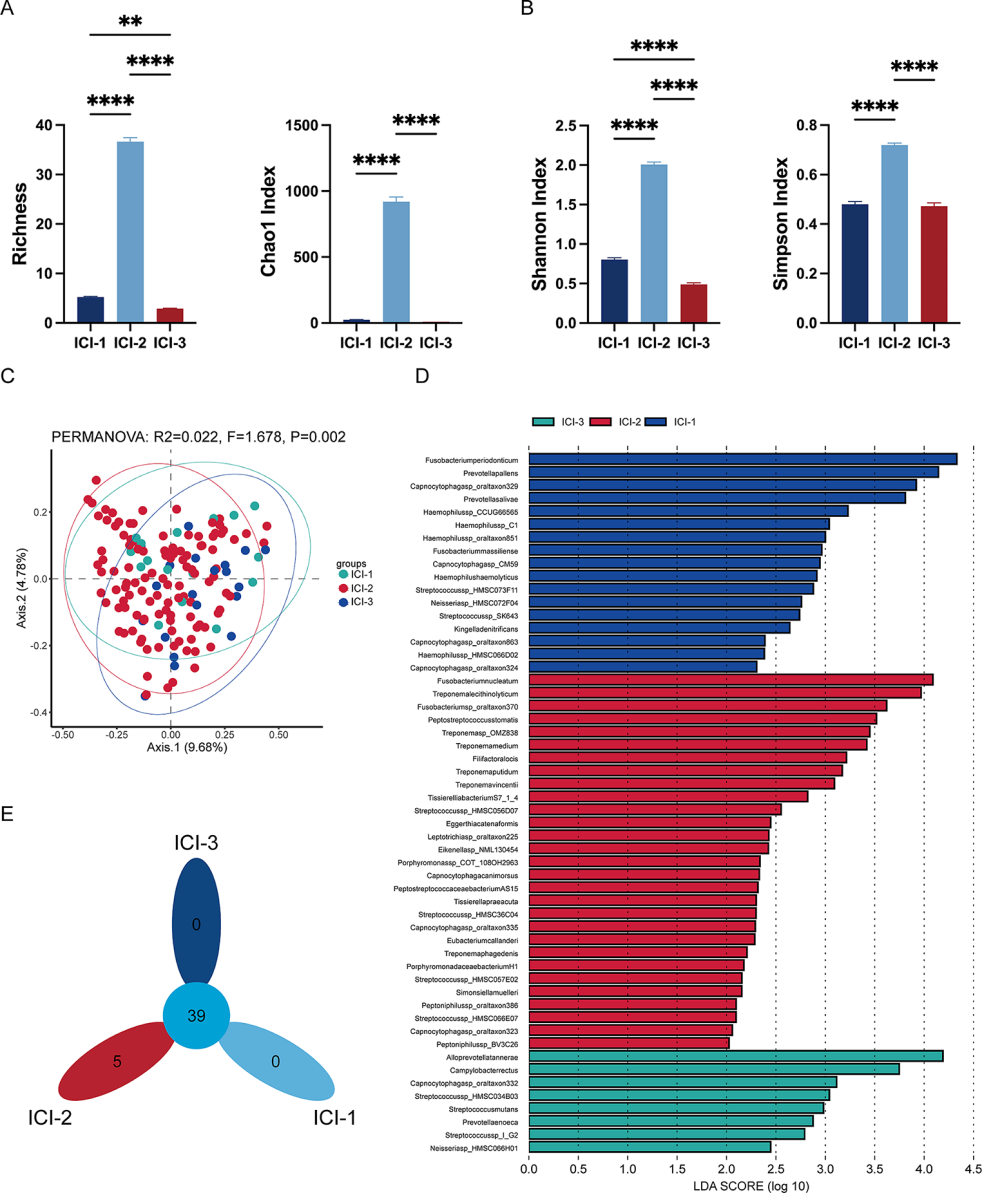
Immune-Related Microbial Features of ICI subtypes. (**A**) Bar Chart of Microbial Richness and Chao1 Index in ICI Subtypes. (**B**) Bar Chart of Shannon Index and Simpson Index in ICI Subtypes. (**C**) Principal Coordinate Analysis (PCoA) of ICI Subtypes in HNSCC. Each point in the plot corresponds to a sample, grouped, and color-coded by subtype – ICI-1, ICI-2, and ICI-3. The plot is annotated with the results of a multivariate statistical test, showing an R-value of 0.022 and an F-statistic of 1.678, with a significant p-value of 0.002. (**D**) LEfSe Analysis of Microbial Differences Among ICI Subtypes. Each bar in the plot represents a microbial feature, with its length indicating the LDA score, set at a threshold greater than 2 for significance. The bars are color-coded to represent different ICI subtypes: blue for ICI-1, red for ICI-2, and green for ICI-3. (**E**) Petal Plot of Specific Microbial Species in ICI Subtypes. Each ‘petal’ in the plot represents a different microbial species, with the size and number of petals corresponding to the number of unique species within each ICI subtype

To investigate whether microbes influence the three immune subtypes, we examined the microbial characteristics across these subtypes. Compared to ICI-1 and ICI-3, the ICI-2 subtype exhibited an increased microbiota richness and diversity (Fig. 3A and B). Furthermore, beta diversity analysis using Principal Coordinates Analysis (PCoA) demonstrated subtle but significant alterations in the microbial community structure within the ICI subtypes (PERMANOVA *p* < 0.05) (Fig. 3C).Utilizing Linear Discriminant Analysis Effect Size (LEfSe), we analyzed the differential microbial composition at species levels among the three immune phenotypes. In the LEfSe analysis, several features were identified as significantly differentiating between the groups (Fig. 3D). Notably, *Fusobacterium nucleatum* exhibited the highest Linear Discriminant Analysis (LDA) score of ICI-2, indicating a strong association with ICI-2. Regarding *Fusobacterium periodonticum* and *Alloprevotella tannerae*, they were identified as having the highest LDA scores in ICI-1 and ICI-3, respectively. In the ICI-2 subtype, we identified five uniquely expressed microbial species: *Capnocytophaga canimorsus*, *Tissierellia bacterium S7-1-4*, *Simonsiella muelleri*, *Porphyromonadaceae bacterium H1*, and *Streptococcus sp. HMSC056D07*(Fig. 3E).

### Correlation between immune-related microbial features and ICI patterns

**Fig. 4:**
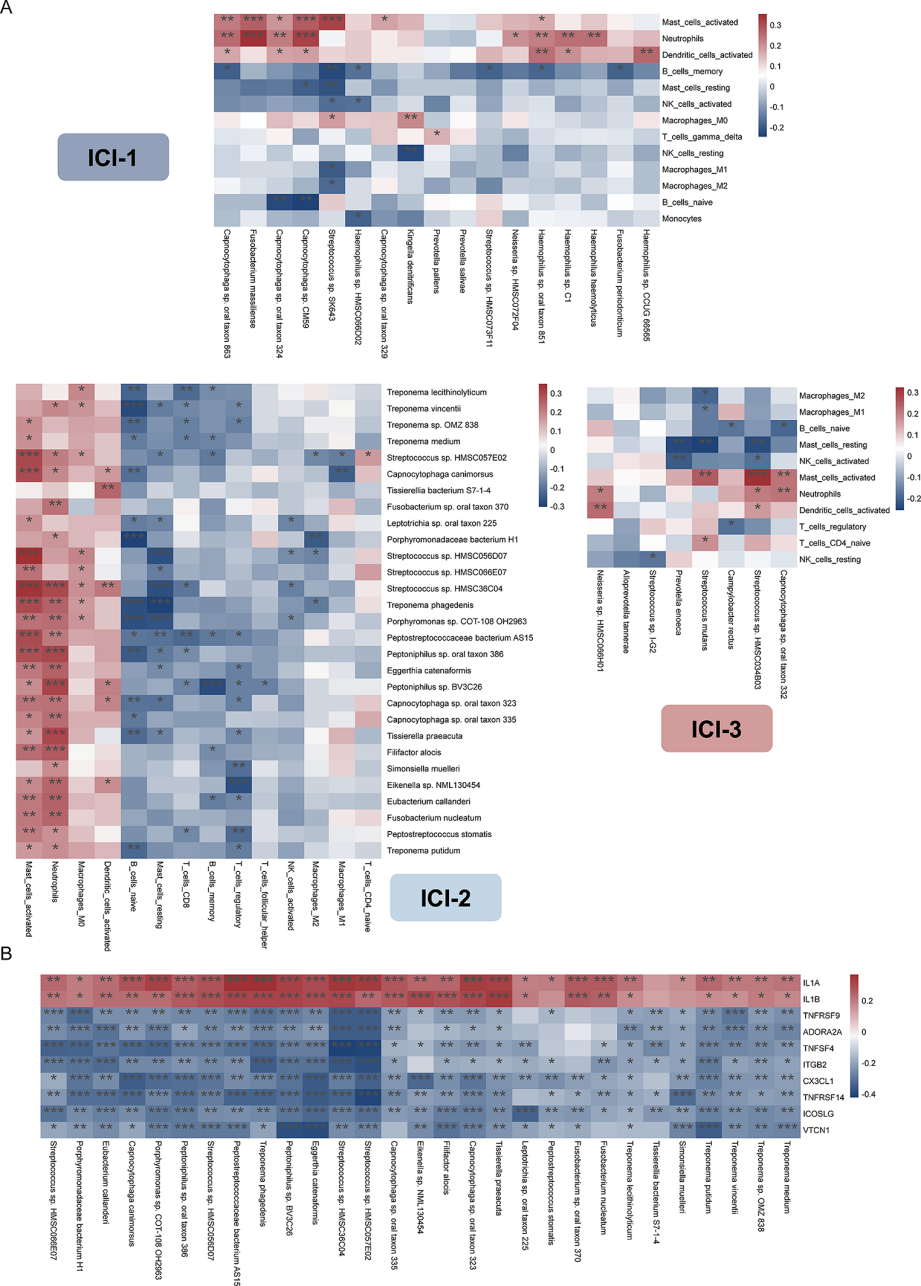
Correlation Between Immune-Related Microbial Features and ICI Patterns. (**A**) Heatmap of Correlations Between ICI-2-Related Microbes and Immune Cells. Each row represents a specific microbe, while each column corresponds to a type of immune cell. (**B**) Heatmap of Correlations Between ICI-2-Related Microbes and Immune-regulatory genes. Each column represents a specific microbe, while each row corresponds to a type of immune cell. The color intensity within the heatmap indicates the strength and direction of the correlation: warm colors signify positive correlations, and cool colors indicate negative correlations. *p < 0.05, **p < 0.01, ***p < 0.001

We further study the association between specific microbial features and their corresponding immune subtypes. Utilizing the LEfSe algorithm, our analysis revealed that in the ICI-2 subtype, a majority of the significantly enriched bacteria were positively correlated with activated mast cells and neutrophils, though there were notable exceptions (refer to Fig. 4A for comprehensive details). Moreover, an array of bacteria, including *Treponema lecithinolyticum*, *Treponema vincentii, Treponema sp. OMZ 838*, *Treponema medium*, *Streptococcus sp. HMSC36C04*, *Peptostreptococcaceae bacterium AS15*, *Peptoniphilus sp. oral taxon 386*, *Peptoniphilus sp. BV3C26*, and *Peptostreptococcus stomatis*, exhibited a significant negative correlation with CD8 + T cells. Additionally, our findings indicate a substantial negative correlation between activated NK (Natural Killer) cells and specific bacteria such as *Leptotrichia sp. oral taxon 225*, *Streptococcus sp. HMSC056D07*, *Streptococcus sp. HMSC36C04*, and *Porphyromonas sp. COT-108 OH2963*. The microbial enrichment in ICI-1 shows a correlation with immune cells similar to that observed in ICI-2. However, in ICI-3, the uniquely expressed microbes do not exhibit a significant correlation with CD8 T cells. Apart from *Tissierellia bacterium S7-1-4*, all microbial species enriched in ICI-2 exhibited a significant positive correlation with IL-1 (Fig. 4B).

### Performance of an immune-related microbiome based neural network prognosis model

**Fig. 5:**
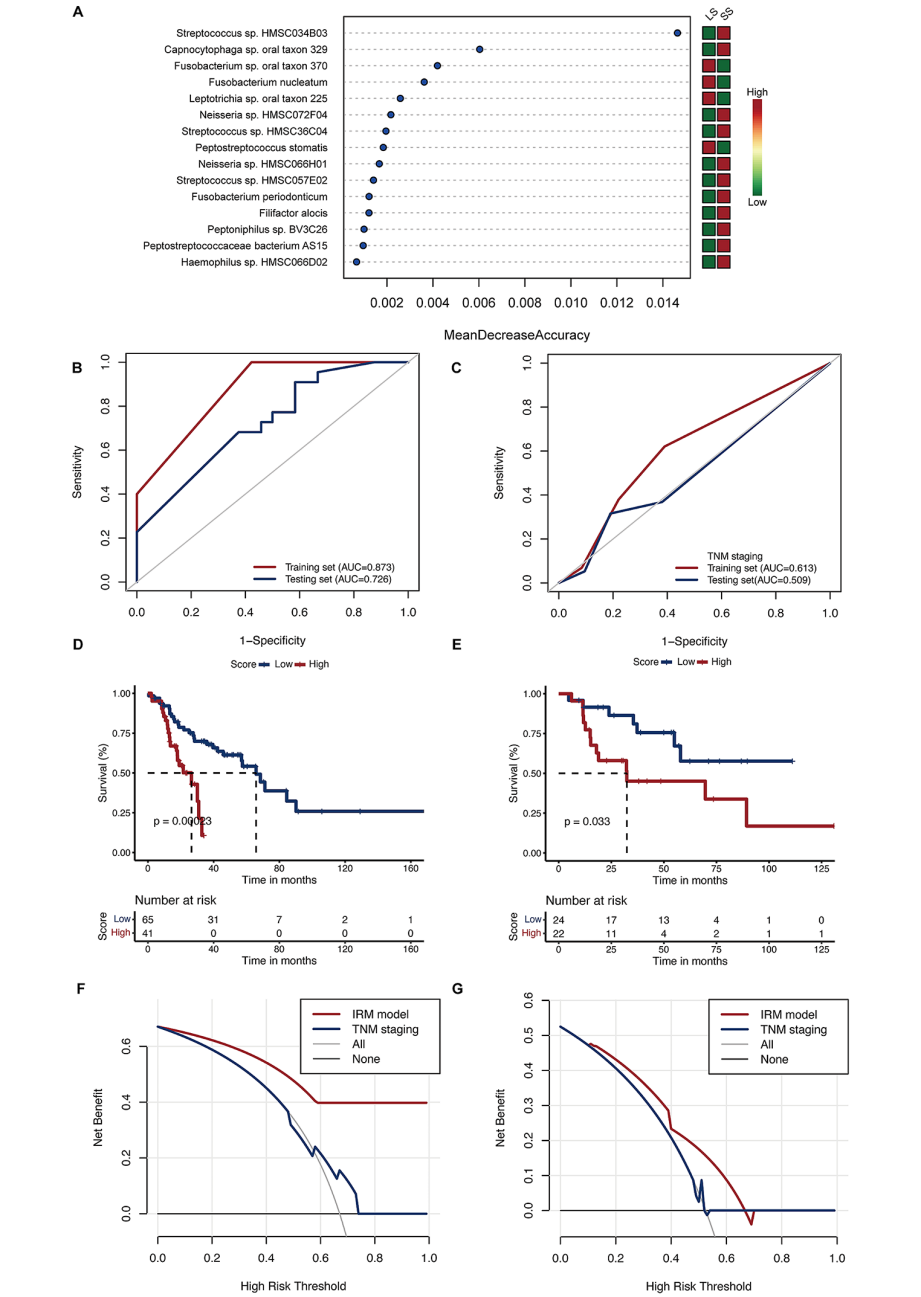
Performance of An Immune-Related Microbiome Based Neural Network Prognosis Model. (**A**) Random Forest Feature Selection for Immune-Related Microbial Features. The x-axis represents the Mean Decrease in Accuracy, indicating each feature’s importance in the model, while the y-axis lists the top 15 immune-related microbial features. The plot is divided into LS and SS to compare the relative importance of these features in predicting patient survival outcomes. (**B**) ROC Curve Analysis of the IRM Model for Survival Prediction in HNSCC. The ROC curve for the training set is shown, with an AUC of 0.873 (95%CI: 0.824–0.922). The AUC of testing set is 0.726 (95% CI :0.588–0.864). (**C**) ROC Curve Analysis of the TNM Staging Model for Survival Prediction in HNSCC. The ROC curve for the training set is shown, with an AUC of 0.613 (95% CI: 0.496–0.729). The AUC of testing set is 0.509 (95% CI: 0.348–0.669). (**D, E**) Kaplan-Meier Survival Curves for IRM Model in Training Set (**D**) and Testing Set (**E**). The survival probabilities over time are plotted, with the red line representing patients categorized with a high risk score and blue line representing low risk score. (**F, G**) Decision Curve Analysis of the IRM and TNM Models for HNSCC in Training Set (**F**) and Testing Set (**G**). The y-axis represents the net benefit, quantifying the true positive rate against the false positive rate, while the x-axis indicates the threshold probability

Subsequently, we investigated whether the microbial features associated with immune subtypes could predict the prognosis of HNSCC patients. Initially, based on OS, we categorized patients into two groups: long-term survival (survival of three years or more) and short-term survival (less than three years). From 54 immune-related microbial species, the most discriminative features for predicting survival length were identified using a random forest (RF) algorithm (Fig. 5A). Features were selected as model variables based on two criteria: a MeanDecreaseAccuracy greater than zero and a p-value less than 0.05. Six immune-related microbial features met these criteria, including *Streptococcus sp. HMSC034B03*, *Streptococcus sp. HMSC36C04*, *Neisseria sp. HMSC066H01*, *Streptococcus sp. HMSC057E02*, *Haemophilus sp. HMSC066D02*, and *Peptoniphilus sp. BV3C26*. Subsequently, we employed the identified immune-related microbial features to construct a prognostic model using a neural network algorithm. In the training set, this prediction model demonstrated significant discriminatory power in differentiating between long-term and short-term survival, as evidenced by an area under the receiver operating characteristic (ROC) curve of 0.873. In the testing set, the model achieved an AUC of 0.726 (Fig. 5B).

To evaluate whether our model outperforms traditional clinical models, we constructed a TNM staging-based logistic regression model. The AUC for the TNM staging model was 0.613 in the training set and 0.509 in the testing set (Fig. 5C). In comparison, our immune-related microbiome (IRM) model demonstrated significantly superior performance over the TNM staging model. Additionally, we conducted time-dependent ROC analysis for the IRM model. The 3-year and 5-year prediction AUCs in the training set were 0.859 and 0.845, respectively. In the testing set, these values were 0.762 and 0.653 (Figure S1A, S1B). These results significantly outperform the TNM staging model, which achieved AUCs of 0.663 and 0.634 in the training set for the same time points (Figure S1C, S1D). Kaplan-Meier survival analysis revealed that the model was able to significantly distinguish between different prognoses in both the training and validation sets. Patients with higher model-predicted scores were associated with poorer prognosis (*p* < 0.05) (Fig. 5D and E). The decision curve analysis showcases the clinical utility of the IRM model by illustrating its net benefit in comparison to the extreme strategies of treating all or none of the patients. This analysis also includes a comparison with the TNM staging model (Fig. 5F and G).

Our IRM model demonstrates significant clinical value not only in predicting OS but also in assessing Progression-Free Survival (PFS). We observed that patients with higher risk scores, as indicated by the IRM model, tend to have shorter PFS (Figure S2A). This finding is crucial for early patient screening and facilitating more timely and targeted therapeutic interventions. Additionally, in our drug sensitivity assessment, patients categorized as high-risk showed higher estimated IC50 values for cisplatin and sorafenib compared to those with lower risk scores (Figure S2B, S2C). This suggests that patients with lower risk scores may exhibit greater drug sensitivity and better chemotherapy response. The collective findings from our study underscore the potential of using immune-related microbial features as prognostic biomarkers in HNSCC.

## Discussion

Recent studies have highlighted the pivotal role of tumor-infiltrating immune cells within the TME in influencing tumor progression and clinical prognosis. These cells are increasingly recognized as valuable targets for therapy [21]. Consequently, strategies aimed at remodeling the tumor-immune microenvironment are emerging as promising approaches to augment the anti-tumor immune response [22]. However, the characteristics of immune phenotyping and the underlying mechanisms remain to be elucidated.

In this study, we integrated the immune cell landscape of HNSCC and categorized patients into three groups based on their immune characteristics. The ICI-1 was characterized by a particularly high stromal fraction, especially in terms of fibroblast proportion. The ICI-2, with the lowest immune scores and the poorest prognosis, was often considered to represent ‘cold tumors.’ In contrast, the ICI-3 had the highest immune scores and the best prognosis, typically referred to as ‘hot tumors.’ We observed that within the TME, the ICI-2 subtype, characterized by lower proportions of CD8 T cells, memory B cells, Tfh cells, activated CD4 memory T cells, Treg cells, and naive B cells, exhibited a higher hypoxia score. Additionally, this subtype showed a relatively higher proportion of exhausted T cells. These findings suggest that intratumoral hypoxia may further exacerbate immune suppression. Recent studies indicated that the impact of hypoxia on the tumor immune microenvironment primarily affects the function and distribution of immune cells [23]. Hypoxic conditions, through the activation of factors like HIF-1, can alter the activity of immune cells such as T cells and macrophages, diminishing their anti-tumor effects in the TME [24, 25]. Additionally, hypoxia may induce phenotypic changes in tumor-infiltrating immune cells, for instance, promoting the accumulation of immunosuppressive cells like regulatory T cells (Tregs) and myeloid-derived suppressor cells (MDSCs), thereby inhibiting the immune system’s attack on the tumor [26, 27]. These alterations collectively facilitate tumor immune evasion and can adversely affect the efficacy of cancer treatments.

A mounting amount of research suggests that the microbiome is essential for modifying immune responses to cancer treatment [28]. Within the DEGs between ICI-2 and ICI-3, we noted that genes upregulated in ICI-2 were enriched in pathways related to bacterial infection of epithelial cells. This finding suggested that microbes in ICI-2 were involved in modulating the immune system. The microbial species richness and diversity in ICI-2 were significantly higher compared to the other two immune subtypes. We identified 17 species that were relatively enriched in ICI-1, 29 in ICI-2, and 8 in ICI-3. Notably, five microbial species were found to be specifically expressed in ICI-2. Among these five specific microbial species, *Porphyromonadaceae bacterium H1* warrants particular attention. Previous studies have demonstrated an increased abundance of some members of the *Porphyromonadaceae* family in colorectal cancer, which may influence the TME and relate to the tumor’s immune response [29]. The role of these bacteria in the TME could involve altering the distribution of immune cells within the tumor and promoting tumor cell growth [30]. However, research in this area is ongoing, and the specific mechanisms by which *Porphyromonadaceae* bacteria affect tumor development and treatment are not yet fully understood.

The advantages of machine learning in selecting microbial features are primarily manifested in its ability to process and analyze large-scale, complex microbiome datasets. This approach identifies microbial biomarkers associated with specific health conditions or diseases. Machine learning efficiently handles the high dimensionality and complexity of microbiome data, uncovering intricate interactions between microbes and their hosts [31]. Furthermore, machine learning models can predict disease risk or treatment responses by learning patterns in the data, thereby supporting personalized medicine. For instance, in cancer research, machine learning is utilized to analyze the relationship between gut microbiomes and cancer progression [32]. It is also used to identify microbial communities associated with oral diseases [33]. These applications demonstrate the immense potential of machine learning in microbiome research. In this study, we initially employed a random forest algorithm to select immune-related microbial features with predictive value for differentiating long-term and short-term survival. Subsequently, using these six microbial features, we constructed a predictive model utilizing a neural network algorithm. The robust performance of our IRM model, particularly in comparison to the traditional TNM staging model, highlights the value of integrating microbial data into cancer prognosis. The significant predictive accuracy in both short-term and long-term survival predictions, validated across training and testing datasets, reinforces the model’s relevance in clinical settings. Moreover, the decision curve analysis confirms the practical advantage of the IRM model, suggesting its efficacy in guiding treatment decisions more effectively than conventional methods. These results pave the way for a more nuanced understanding of HNSCC prognosis and potentially open new avenues for personalized treatment strategies that consider the unique microbial composition of each patient’s tumor. Ultimately, the integration of multi-omics data, including microbial features, into clinical practice could significantly enhance patient management and treatment outcomes in head and neck cancer.

This study has several limitations. For instance, as indicated by the transcriptome data analysis, a single taxon might show associations with different immune-cell subtypes in conflicting ways. Secondly, we were unable to delineate the functional characteristics of each immune-related microbe and their interaction with immune cells. To validate the role of microbes more conclusively in affecting immune-cell infiltration, further in vitro and in vivo experiments are necessary.

In conclusion, this study offers a comprehensive exploration of the ICI landscape in HNSCC. We provide a detailed scenario of immune regulation in HNSCC and, for the first time, report a correlation between differing ICI patterns, the intratumor microbiome, and patient prognosis. This research aids in identifying prime candidates for optimizing treatment strategies in HNSCC.

### Electronic supplementary material

Below is the link to the electronic supplementary material.

Supplementary Material 1

Supplementary Material 2

## Data Availability

Publicly available datasets were analyzed in this study. These data can be found here: https://www.cancer.gov/tcga. The microbiota date can be found on TCMA database (https://tcma.pratt.duke.edu).
