# Supplementary figures and images for: Multi-omics analysis of immune-related microbiome and prognostic model in head and neck squamous cell carcinoma
Source: Clin Oral Investig. 2024 Apr 20;28(5):263. doi: 10.1007/s00784-024-05645-y (PMC11032295; doi:10.1007/s00784-024-05645-y)

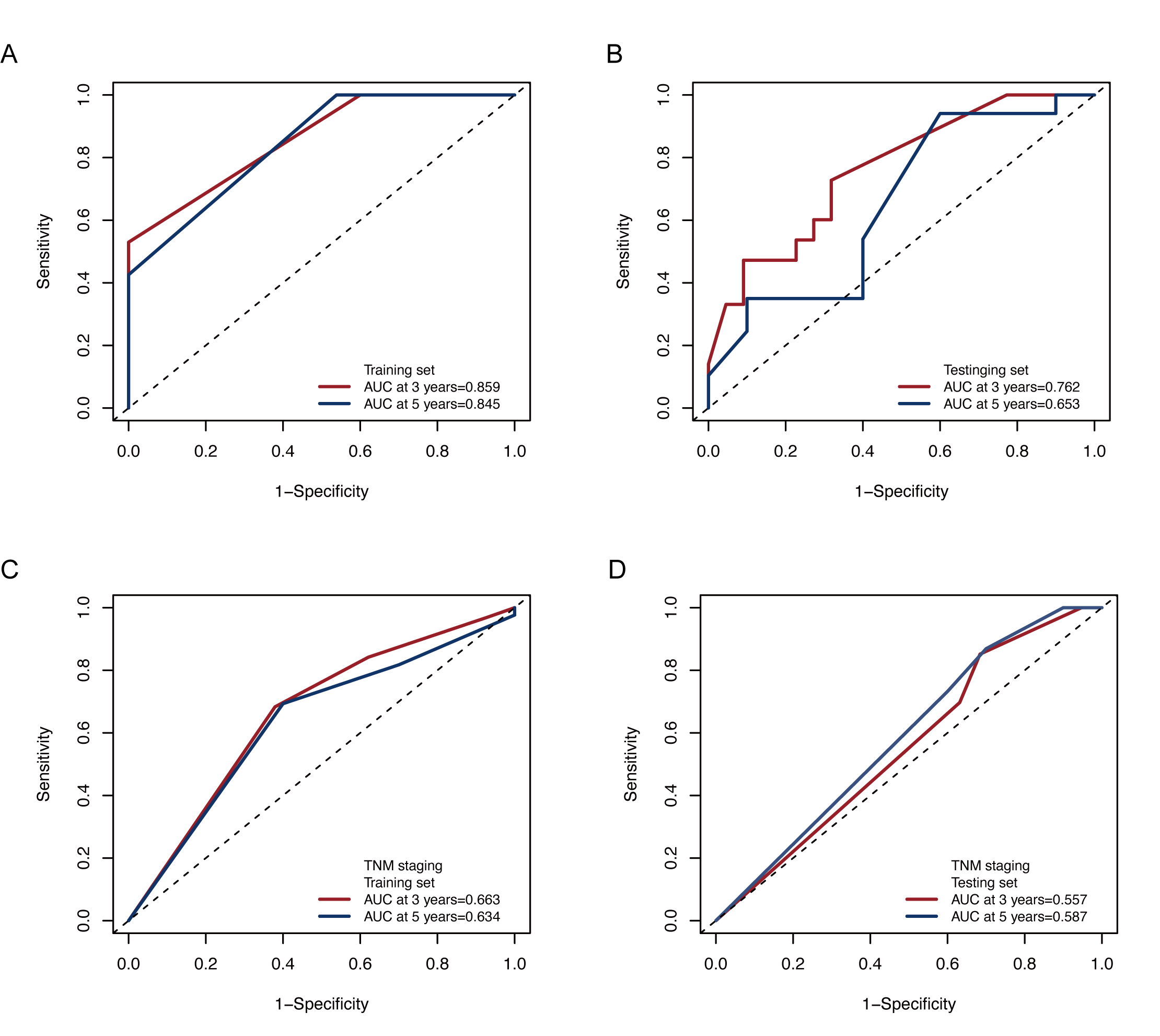

Supplement: Supplementary file 1 — Supplementary Material 1 [file 784_2024_5645_MOESM1_ESM.tif]

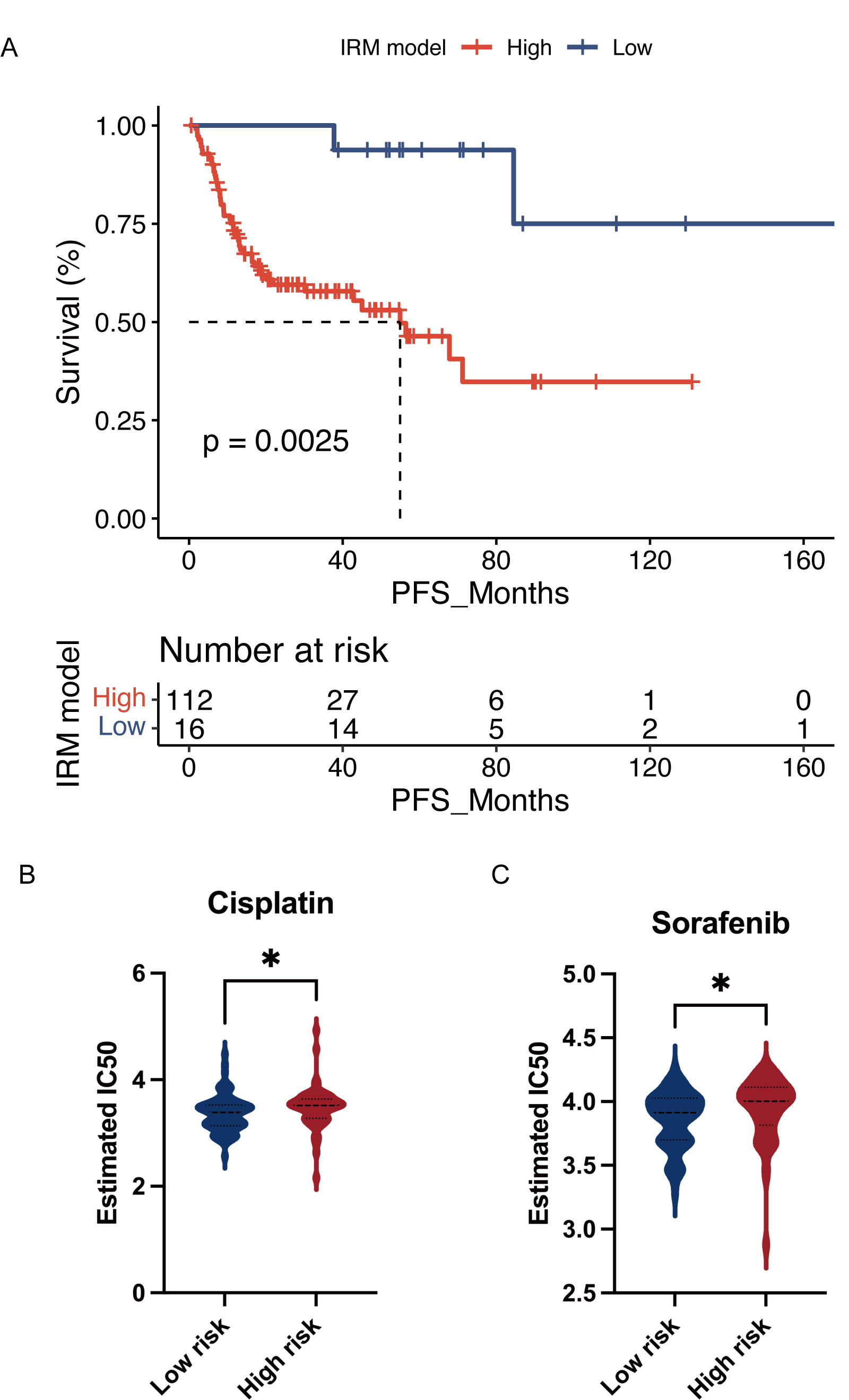

Supplement: Supplementary file 2 — Supplementary Material 2 [file 784_2024_5645_MOESM2_ESM.tif]
